# Kerogen Swelling and Confinement: Its implication on Fluid Thermodynamic Properties in Shales

**DOI:** 10.1038/s41598-017-12982-4

**Published:** 2017-10-02

**Authors:** Manas Pathak, Hyukmin Kweon, Milind Deo, Hai Huang

**Affiliations:** 1 Department of Chemical Engineering, University of Utah, Salt Lake City, 84117 Utah USA; 2 Idaho National Laboratory, Idaho Falls, USA

## Abstract

Type I kerogen was isolated from Green River Shale and characterized using SEM, TGA, DSC, and nitrogen adsorption. The swelling behavior of this kerogen with decane was analyzed using traditional test-tube swelling experiment and Dynamic Light Scattering. The TGA and DSC were used to analyze the thermodynamic behavior of decane that was sorbed in the kerogen and show that kerogen suppresses the boiling point of decane due to the effect of confinement. However, the suppression is larger when oil (a multicomponent mixture) was used, possibly due to the combined effect of differential uptake of components by kerogen (kerogen prefers and sorbs polars and aromatics more than saturates, leading to splitting of oil into a sorbed and a free phase) and confinement in nano pores. Test-tube swelling, TGA, and DSC experiments were also performed on pyridine(polar-aromatic)-swelled kerogen. The combined and individual contributions from the two effects (the effect of confinement and differential uptake of hydrocarbon components) on properties of liquid in contact with kerogen, are studied in this work. Molecular Dynamics (MD) simulations revealed the variation in the swelling of type II kerogen in the presence of same amount of different liquids (differential swelling of kerogen).

## Introduction

**Figure 1:**
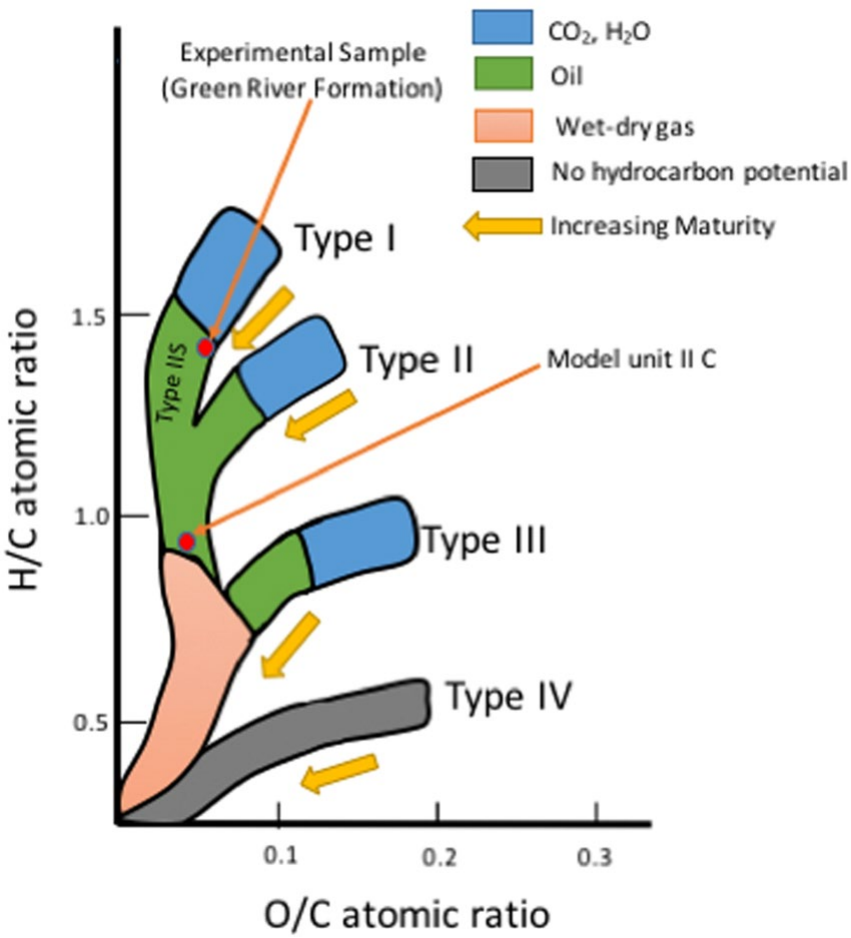
van Krevelen diagram showing the four types of kerogen and how their elemental compositions change as they mature. The red dots show the types of kerogen used in the experiments and molecular dynamics simulations presented in the text. The H/C and O/C ratios of kerogen sample used in experiments are 1.46 and 0.07, respectively based on the elemental composition of kerogen isolated from Mahogany zone, Green River Formation in a previous work^24^. The H/C and O/C ratios of molecular model of kerogen (unit II C from Ungerer) are 0.905 and 0.054, respectively. The plot is re-drawn after the work of McCarthy *et al*.^25^.

Kerogen is a complex heterogeneous carbonaceous material, and it is the precursor of oil and gas found in the sub-surface. It is the polymeric material formed by the biogeochemical alteration of detrital and dissolved organic matter that was deposited together with inorganic sediment, and it is the primary source of oil and gas accumulations throughout the world. Kerogen is a key component of shale rocks and plays a key role in the storage and recovery of hydrocarbons from them. Current techniques for the recovery of hydrocarbons from shales leave over 80% of hydrocarbons in the subsurface^1^. Kerogen is the fraction of the organic matter in buried sediments that is insoluble in common solvents. Although kerogen does not dissolve in organic solvents, like insoluble synthetic polymers such as elastomers and other porous materials^2^, it sorbs and is swollen by them. Kerogen is usually classified into four types based on the depositional environment, the biological source of the organic matter from which it was derived and its elemental composition, particularly the H/C and O/C ratios. Type I (predominantly lacustrine) and type II (predominantly marine) kerogens have high H/C and low O/C ratios, and they are capable of generating oil and gas as progressive burial in the subsurface increases their temperature and pressure. Type III (predominately terrestrial) humic kerogen has lower H/C and higher O/C ratios than types I and II. It generates natural gas but little or no oil. Type IV kerogen is the recalcitrant organic matter that has been pyrolyzed, oxidized and/or recycled. It has the lowest H/C and highest O/C ratios of any type of kerogen, and it cannot generate significant amounts of oil or gas. A fifth class, type IIS, sulfur-rich type II kerogen, with >8% weight sulfur, is often included in this kerogen classification scheme (Fig. 1). Both type I and type II kerogens show swelling in the presence of hydrocarbon fluids based on several previous studies^3–11^. Evidence for the hypothesis that the thermodynamic properties of sorbed hydrocarbon liquids that swell kerogens are different has been provided in the paper. This effect could be referred to as ‘effect of swelling of kerogen’ or ‘effect of differential uptake by kerogen’ or ‘effect of preferential sorption by kerogen’ on pressure-volume-temperature (PVT) properties of hydrocarbon fluids in contact with kerogen. This effect occurs because of preferential sorption of some hydrocarbon components by kerogen leading to a phase split between sorbed oil and free oil phases. The sorbed oil has different PVT properties compared to the free oil. The effect of swelling of kerogen is modeled for Eagle Ford shale and oil in a recently published article^12^ by the authors, however, the current work goes a step beyond to experimentally prove this effect. This effect is different from the previously well-studied^13–22^ effect of confinement of fluids residing in the nano pores of the kerogen, on their PVT properties. The effect of confinement is studied in another recent article^23^ by the authors and the current work aims to experimentally prove and differentiate it from the effect of swelling. Experiments using analytical tools like Differential Scanning Calorimeter (DSC) and Thermogravimetric Analyzer (TGA) were designed in the current work to prove the two effects and understand their combined and individual contributions in changing the PVT properties of liquids in nano porous, kerogen-rich shale rocks.

**Figure 2:**
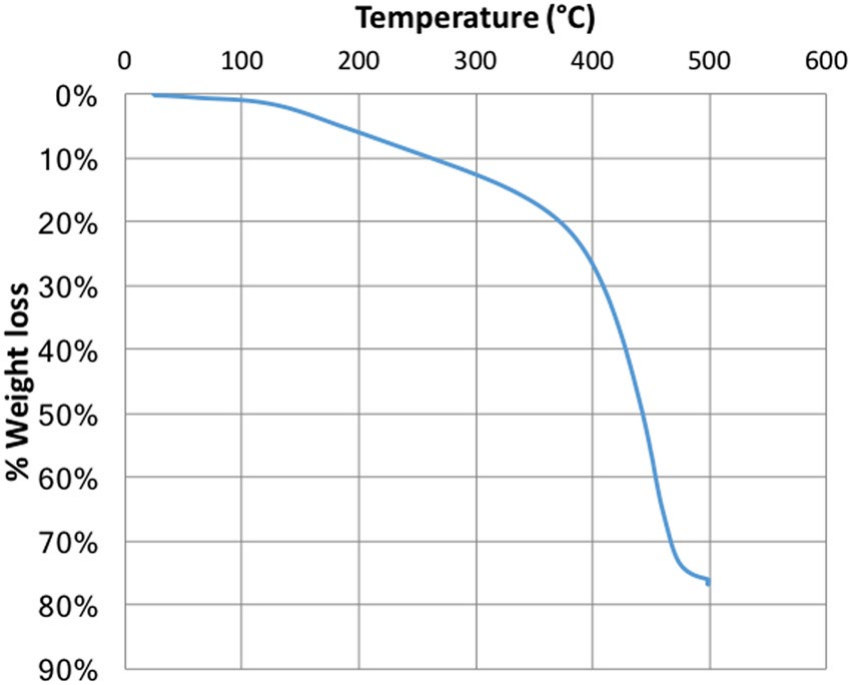
TGA data showing the weight loss curve of kerogen and that the isolated kerogen consists of over 75% organic matter.

**Figure 3:**
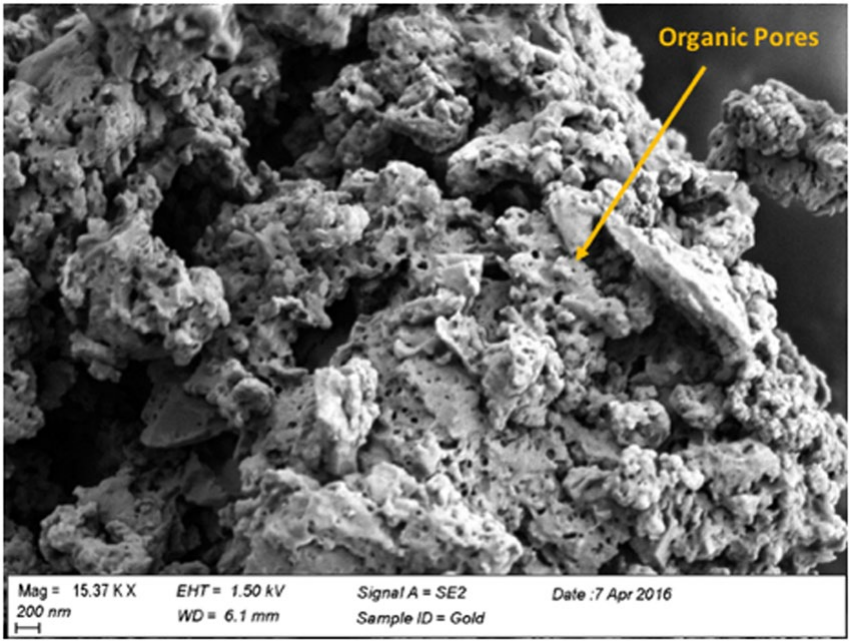
Scanning Electron Microscopy (SEM) image showing presence of sub-micron pores in isolated kerogen of original mesh size of 100 µm. Zeiss SEM (SIGMA 500) was used to obtain the image.

**Figure 4:**
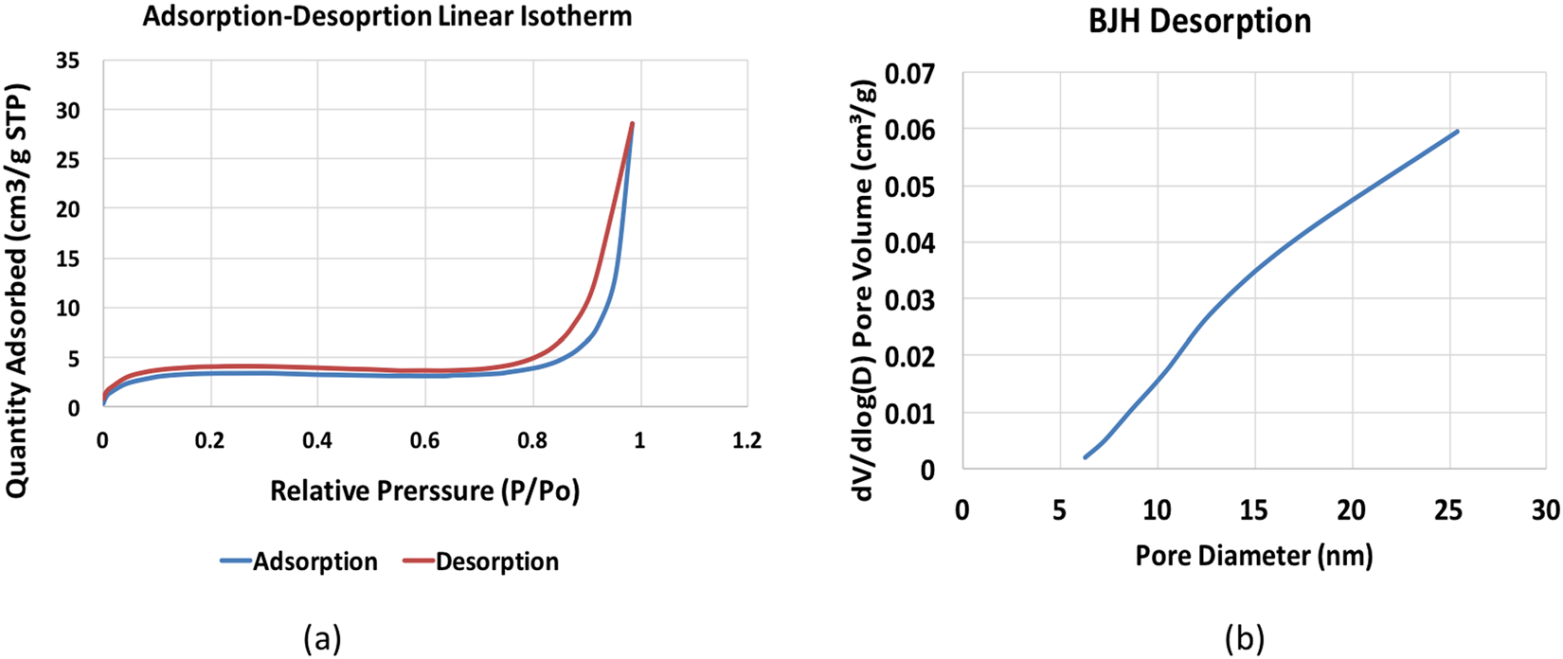
(**a**) Nitrogen adsorption-desorption isotherm in the left shows a hysteresis loop throughout the relative pressure range. (**b**) The Figure on right shows the mean pore diameter of 17.7 nm in the BJH desorption.

Type I kerogen was isolated from the Green River Formation shale sample by series of acid treatments based on previous kerogen isolation procedures^26–30^ and used in the experiments reported here. The kerogen was extracted from samples collected from the Mahogany zone of Green River Formation, provided by Utah Geological Survey. The average organic matter in powdered oil shales was estimated to be 11.5% of the total weight of oil shale^31^ sample. Based on elemental analysis performed in a previous work^24^ on shale samples from Mahogany zone, this kerogen falls in the Type-1 category on van Krevelen classification diagram. This isolated kerogen sample was analyzed using a scanning electron microscope (SEM) and thermogravimetric analyzer (TGA). In practice, it is not possible to completely separate the organic matter from the inorganic matter in organic-rich shales. In this case, the TGA data (Fig. 2) shows that the isolated kerogen consists of more than 75% organic matter by weight. GC-MS analysis of the toluene used to wash the isolated kerogen demonstrated that there was no dissolvable organic bitumen in the kerogen. The SEM image shown in Fig. 3 indicates that the diameters of the pores in the isolated kerogen are on the order of nano-meters as reported previously^32–36^. The nitrogen isotherm (Fig. 4a) of the isolated kerogen has a hysteresis over the relative pressure range of 0.1 to 1.0 (mmHg/mmHg). The Brunauer–Emmett–Teller (BET)^37^ specific surface area of the isolated kerogen is 13.368 m²/g, and the mean pore size calculated by Barret-Joyner-Halenda (BJH)^38^ method from desorption data is 17.7 nm (Fig. 4b).

These nano pores seen in Fig. 3 constitute organic porosity in shales. These pores are primarily generated during the conversion of solid kerogen into oil and gas. While some of the generated hydrocarbon fluids stay in these nano pores, some are sorbed back and some were never expelled out from the kerogen matrix. Such fluids inside the kerogen matrix (partly made up of indistinguishable sorbed fluids and retained fluids) would be called sorbed fluids from here on, for the purpose of this manuscript. The sorbed fluids stay in multi-component equilibrium with free but confined fluids in the nano pores^5,12^. The sorbed fluids cause a net expansion in the volume of kerogen. Such swelling in kerogen is much like swelling of coal with the exception that kerogen swelling has non-specific molecular interactions^5^ (molecular interactions mainly caused by dispersion forces^39^). The hydrocarbons that are not sorbed stay in the form of free but confined fluids in the organic nano pores under the influence of dominant pore-wall – fluid interactions^23^. At nano scale, the surface forces between solid pore-wall and fluids in pores are dominant as surface area per unit volume is significant at the nano scale. These pore-wall – fluid interactions change the behavior of fluids confined in nano pore as discussed in a recent article^23^ published by the authors. In that paper, authors have found that the critical properties of fluids are suppressed in the confined spaces due to dominant interactions with the pore walls. The sorbed hydrocarbons that swell kerogen have different thermodynamic properties than the free but confined hydrocarbons because kerogen preferentially sorbs polars and aromatics more than saturates leading to the change in composition of sorbed oil^5,12^. Thus, the fluid thermodynamic properties of both, confined free oil in nano pore and sorbed oil in shales are different than the bulk oil due to the effect of confinement and swelling of kerogen, respectively. It is, therefore, important to calculate *in-situ* PVT properties of oil in shales taking into account the two effects in order to predict correct volumes and rates of recovery. Abnormal Gas-Oil ratios (GORs)^40–42^ seen in Permian and Eagle Ford Shale plays could be attributed to the change in the *in-situ* bubble points of oil in shales due to the two effects discussed in this work. Sorbed oil may consist of a portion of absorbed fluids and adsorbed fluids (Adsorption is the surface phenomena involving the surface of the solid, while absorption is a bulk phenomenon involving the volume of a permeable solid. Sorption refers to the combined phenomena of absorption and adsorption). The absorbed fluid may stay in equilibrium with adsorbed fluid. However, this absorbed-adsorbed oil interaction is included while characterizing the sorbed fluids through experiments in the current work, as sorbed oil represents the combination of both the fractions. Thus, only the experiments on sorbed fluids is discussed in this work.

Further experiments were conducted on a binary mixture of kerogen and decane to investigate the change in the thermodynamic properties of the sorbed decane. Swollen kerogen samples were prepared by equilibrating kerogen and decane for 24 hours, after which no further swelling was observed, in agreement with previous swelling studies^3–11^. The swelling ratio (Q_v_) is defined as the ratio between the volume of the swollen kerogen and the original volume of the kerogen. Traditional test-tube swelling experiments were performed by recording the height of the kerogen bed in a flat bottom test-tube before and after swelling with decane. Dynamic Light Scattering (DLS) experiments performed on kerogen samples before and after swelling with decane provided a separate independent measurement of the swelling ratio.

At this point, it was hypothesized that TGA and DSC experiments could be performed on kerogen and liquid-swelled kerogen to investigate the two effects. TGA and DSC experiments were performed on kerogen and single component liquid (pure decane) to assess the effect of confinement only, on the phase change of decane as the effect of swelling of kerogen can only be assessed in a multi-component hydrocarbon mixture like oil. The multicomponent mixture allows the kerogen to uptake components to different extents based on preference (polars and aromatics are preferred more than saturates) so that the multicomponent mixture is split into a sorbed and a free fraction^5,12,43–45^. The sorbed fraction has a different composition than the free fraction and is richer in heavier, polars and aromatic compounds. Such fractionation of oil by the kerogen is discussed in details in the recent article^12^ by the authors. This compositional split does not occur in a single component and thus, the TGA and DSC results would only show the effect of confinement in the kerogen-decane sample. It is postulated that the DSC and TGA of kerogen and oil would show both the effects. Thus, the swelling of kerogen occurs with decane but the partitioning, as postulated for multiple components does not occur.

TGA and DSC experiments were also performed on pyridine and pyridine-swelled kerogen to investigate the higher sorption capacity of kerogen for polar-aromatics (pyridine) than for saturates (decane). The use of pyridine reveals the swelling behavior of kerogen with generic polar-aromatic organic liquids as compared to non-polars. The swelling of oil-swelled kerogen and pyridine-swelled kerogen was measured by traditional test-tube swelling experiments as well. DLS swelling experiments were not performed with oil and pyridine because of issues related to the equipment compatibility. The oil was collected from the Frontier Reservoir in Clay Basin Field, Duggett county, Utah. The TGA and DSC experiments were conducted over temperature ranges in which the kerogen is chemically inert on laboratory timescales. These ranges were determined based on the TGA and DSC runs performed for kerogen, and discusssed in the Results section.

Both type I and II kerogen swell based on several previous test-tube swelling studies^3–11^ and further study on type I kerogen was performed in this work. However, in order to gain further insight into the differential swelling behavior of kerogen, Molecular dynamics (MD) simulations were performed in this work. The motivation behind MD swelling simulations was not to replicate the test-tube swelling experiments but to study the swelling of any kerogen that swells (both type I and II swell^3–11^) in order to find a possible reason for change in thermodynamic properties of liquids that sorb into the kerogen. These simulations thus provide some explanation for the differences in results in the DSC experiments conducted on decane and pyridine-swelled kerogen, and oil-swelled kerogen.

MD simulations are also useful since experiments are difficult to perform under pressures that are characteristic of most US shale plays. Based on the elemental analysis, analysis of pyrolysis products and spectroscopic data, Ungerer *et al*.^46^ have developed realistic kerogen models that are used in this work to perform molecular dynamic simulations of kerogen with organic liquids. These simulations are performed at reservoir temperature and pressure using type II kerogen model to simulate its swelling in the presence of 17 organic liquids chosen in this study.

## Results

**Figure 5:**
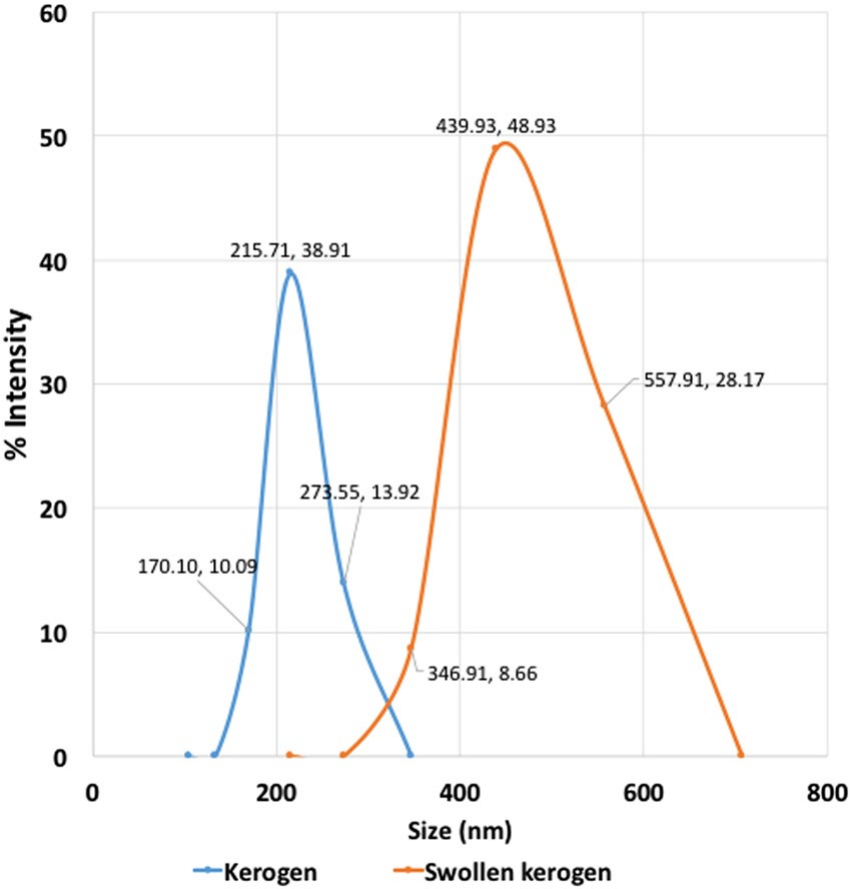
Size distribution for unswollen and swollen kerogen particles obtained from one of the runs of dynamic light scattering (DLS) experiments. The kerogen particles were swollen by equilibration with excess decane for 24 hours. For this particular run, the mean particle size of the kerogen particles was 219.78 nm before swelling and 448.25 nm after swelling. The polydispersity (PdI) = the square of the standard deviation/mean diameter in this case is close to 0.05 indicating a monodisperse sample and suitability of DLS in the size measurement of kerogen particles.

The swelling ratio, Q_v_, for type I kerogen equilibrated with excess decane calculated from the test-tube swelling was 1.23 and is consistent with the study by other authors^5^. The dynamic light scattering (DLS) experiments performed in this study also showed a considerable swelling in kerogen. The DLS works on the principle of estimating the particle diameter by determining the diffusion coefficient in the media (decane in this case) using the Stokes Einstein’s relation. The diffusion coefficient is calculated from the quasi-elastic scattering of light from the suspended particles of the solution of decane and kerogen. Q_v_ for decane-swelled kerogen (=2.0) inferred from DLS is higher than the ratios measured by test-tube experiments possibly because DLS measures the hydrodynamic radius of particles. Hydrodynamic radius of particles is defined as the effective radius of a particle in a solution measured by assuming that it is a body moving through the solution and resisted by the solution’s viscosity. The hydrodynamic radius could include all the liquid molecules attracted to the particle and attached at the surface of the particle. As a result, it is possible for a small particle to have a larger hydrodynamic radius than a large particle – if it is surrounded by more liquid molecules. The DLS experiments were repeated three times, and the mean radii of three runs before and after swelling were equal to 249.46 nm (standard deviation of 21.50 nm) and 505.21 nm (standard deviation of 49.27 nm), respectively. A result from one such run is shown in Fig. 5.

**Figure 6:**
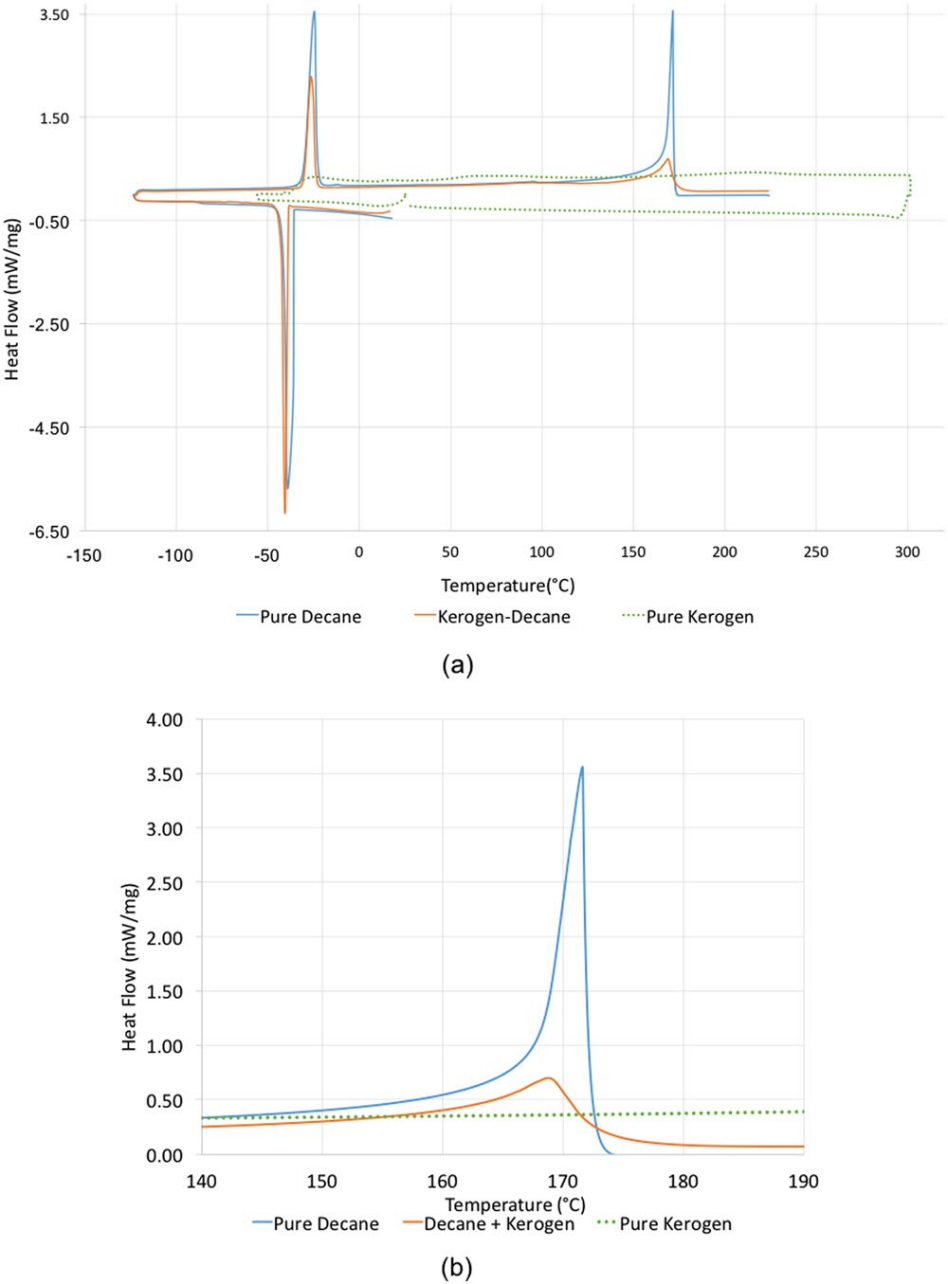
(**a**) DSC data shows that the decane sorbed in kerogen boils, melts and crystallizes at lower temperatures than decane in the bulk state. (**b**) Boiling point peak is enlarged to show the difference between the DSC peak of pure decane and that of decane in kerogen.

Differential scanning calorimetry (DSC) results show different temperatures of phase change for decane compared to decane sorbed in kerogen in the decane-swelled kerogen sample. The temperature of vaporization of decane (shown by the peaks in the heat flow in Fig. 6) was suppressed by the kerogen matrix as indicated by the lower onset temperatures for vaporization. Similarly, the melting and crystallization were suppressed for the decane in kerogen as shown in Table 1. Some decane present in 17.7 nanometer pores (mean radii) of kerogen forms the free but confined fraction of the total decane present in the swollen kerogen sample. The other fraction is decane sorbed in the kerogen matrix. However, there is no clear distinction between decane sorbed by the kerogen matrix and decane in nanometer pores. The kerogen with 17.7 nm avg. pore size gave results of suppression in bubble point temperature of decane (7.8 °C suppression). This suppression is possibly due to the effect of confinement only, as a result of pore-wall – fluid interactions in the nano pores of kerogen. The effect of preferential sorption does not occur in this case as both sorbed and free fraction is decane. The bubble point was assumed to be at the temperature of onset of the endothermic peak associated with vaporization. This onset was determined as the point of intersection of the tangent line at the point of greatest slope on the leading edge of the peak with the extrapolated baseline.

**Table 1:** Peak and Onset temperature of crystallization, melting, and vaporization of pure decane and decane in kerogen.

**Sample**	**Onset of crystallization (**°C**)**	**Peak of crystallization (**°C**)**	**Onset of melting of solid (**°C**)**	**Peak of melting of solid (**°C**)**	**Onset of vaporization (**°C**)**	**Peak of vaporization (**°C**)**
**Pure decane**	−35.8	−38.9	−29.0	−24.5	167.8	171.6
**Pure decane in kerogen**	−39.9	−40.2	−30.2	−25.9	160.0	168.7

**Figure 7:**
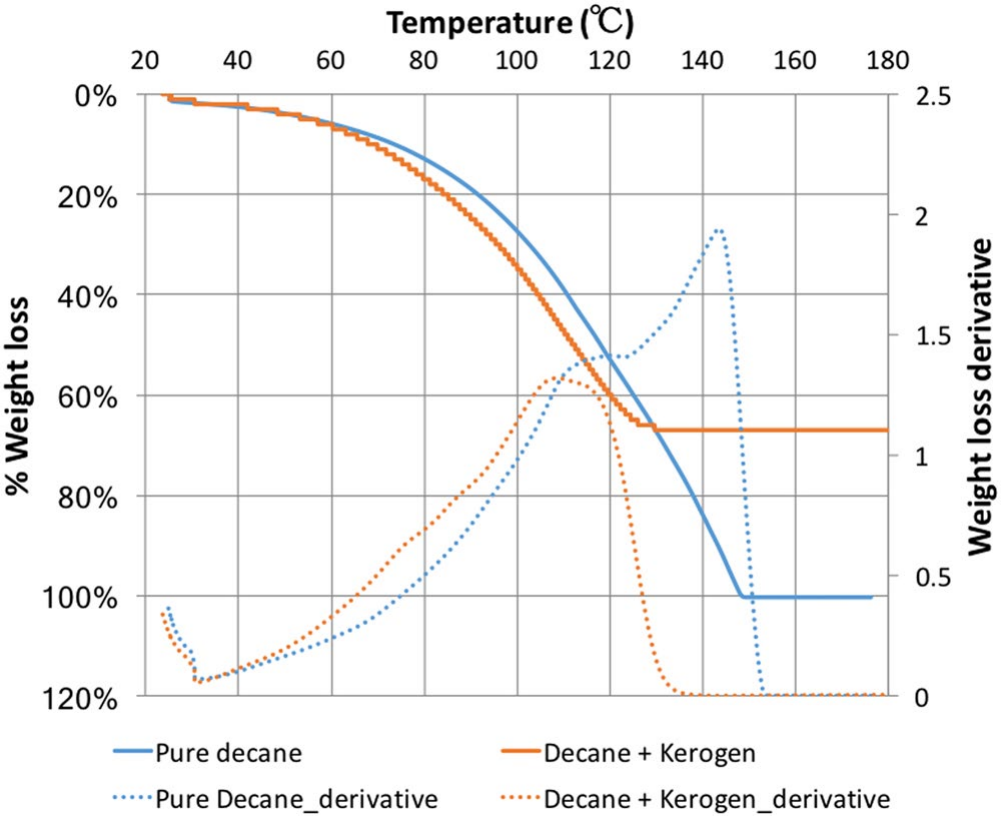
TGA data showing that kerogen interacts with decane and changes its boiling point. The decane-swelled kerogen has 68% decane that was sorbed in the kerogen.

Different weight loss curves were obtained for decane with and without kerogen in TGA. The TGA data showed that the decane sorbed in kerogen was vaporized earlier than the pure decane as seen in Fig. 7. The TGA data also shows that the around 68% of the decane-swelled kerogen was decane, indicated by 68% weight loss in the decane+kerogen sample at temperatures below the boiling point of decane (174.1 °C).

From the differential uptake of liquids by kerogen discussed earlier, it was expected that the DSC conducted on oil (multi-component mixture) and kerogen would largely change the bubble points of the sorbed oil. Such a large change was expected due to combined effect of differential uptake (leading to change in the composition of sorbed oil from the original composition of oil) and confinement of oil in nano pores of kerogen. To confirm this hypothesis, a multicomponent mixture of hydrocarbons such as oil was used to swell kerogen. The kerogen was mixed with Clay Basin oil in test-tube swelling experiments and subsequently, DSC experiments were performed on oil and oil-swelled kerogen. Test-tube swelling experiments revealed that after over 24 hours, the kerogen swelled 1.6 times due to sorption of oil.

**Figure 8:**
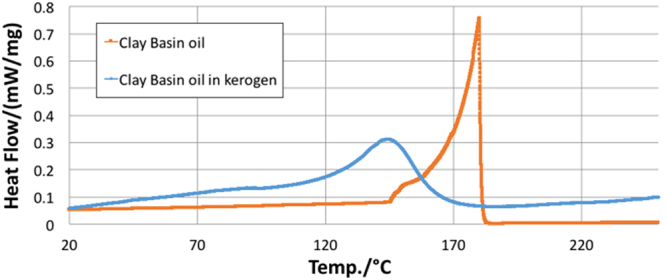
DSC data showing that kerogen sorbed oil and changed the bubble point of the sorbed oil.

It was observed from the DSC experiments on oil and oil-swelled kerogen samples, that a suppression of over 50 °C (Fig. 8) was seen in the bubble point temperature of the oil sorbed in kerogen compared to that of the pure oil (Table 2). This suppression includes the effect of confinement and the effect of preferential sorption (differential uptake) of hydrocarbon components by kerogen. Thus, it is likely that the presence of kerogen changes the thermodynamic properties of fluids due to the effect of confinement of free oil in nano pore and preferential sorption of oil components). The two effects, however, affect bubble point temperature to different extents as suppression in boiling point of sorbed oil (Fig. 8) is more than the suppression measured for decane (Fig. 6). Thus, it is also likely that the effect of preferential sorption changes the PVT properties of oil more than the effect of confinement.

**Table 2:** Temperature of onset and peak of vaporization of Clay Basin oil and Clay Basin oil in kerogen.

**Sample**	**Onset of vaporization (**°C**)**	**Peak of vaporization (**°C**)**
**Oil**	176.1	180.1
**Oil in kerogen**	122.4	143.9

Further experiments were performed with pyridine (a polar-aromatic compound) to investigate the fact that kerogen preferentially sorbs like molecules (polar, aromatic) and hence, exhibits higher swelling with polar-aromatic liquids than with saturates (decane), in case excess liquid is present.

**Figure 9:**
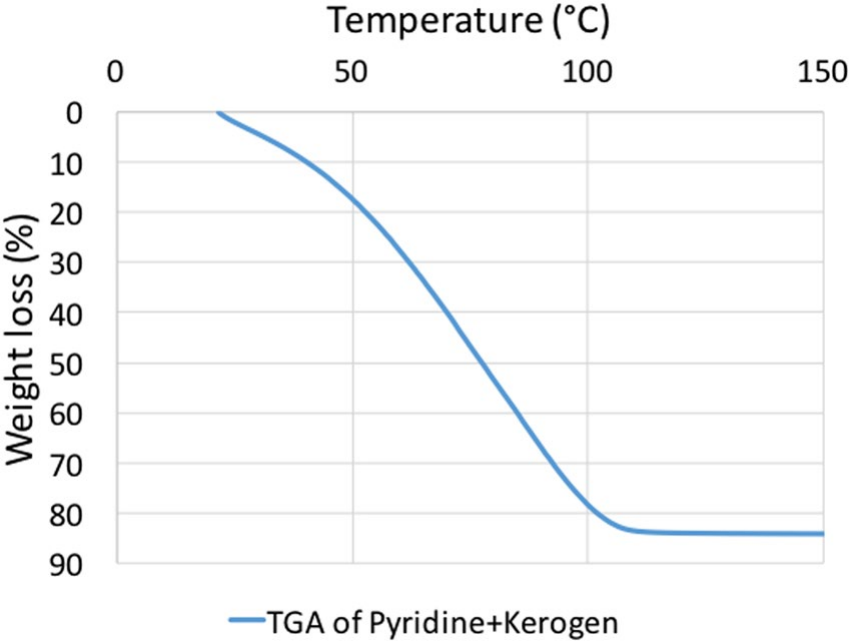
DSC data showing that pyridine-swelled kerogen has 84% pyridine that was sorbed in kerogen. Kerogen sorbs pyridine more than decane (68%) because pyridine has higher solubility in kerogen.

**Figure 10:**
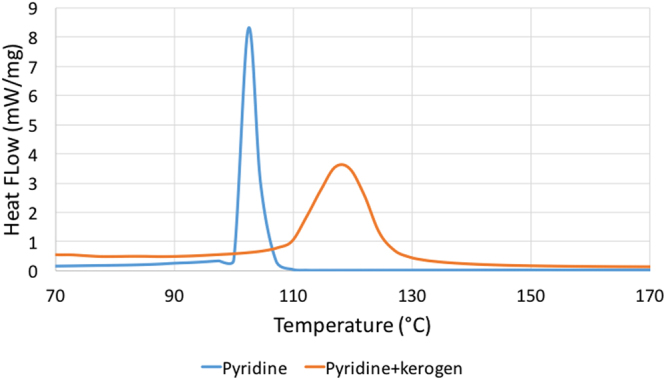
DSC data showing that the boiling point of sorbed pyridine in pyridine-swelled kerogen was elevated by 7.5 °C.

Pyridine swelled kerogen to 1.40 times in 24 hours based on test-tube swelling experiments as recorded previously^5^ as well. TGA experiment on pyridine-swelled kerogen reveals that 84% of the sample was pyridine (Fig. 9). Pyridine, as expected, being a polar liquid was sorbed in larger volume than decane sorbed in kerogen and consequently swelled kerogen more in test-tube swelling experiments where excess liquids are used (Qv of kerogen with decane is 1.23 compared to 1.40 with pyridine in test-tube swelling experiments). However, DSC experiments on pyridine and pyridine-swelled kerogen indicated that sorbed pyridine’s boiling point was elevated as compared to the suppression seen in sorbed decane (Fig. 10 and Table 3). A possible reason for higher boiling point could be that pyridine being polar and aromatic is held stronger (more soluble in kerogen) in kerogen and thus, boils off at higher temperatures.

**Table 3:** Temperature of onset and peak of vaporization of pyridine and pyridine in kerogen.

**Sample**	**Onset of vaporization (**°C**)**	**Peak of vaporization (**°C**)**
**Pyridine**	101.5	103.1
**Pyridine in kerogen**	109.0	118.1

**Figure 11:**
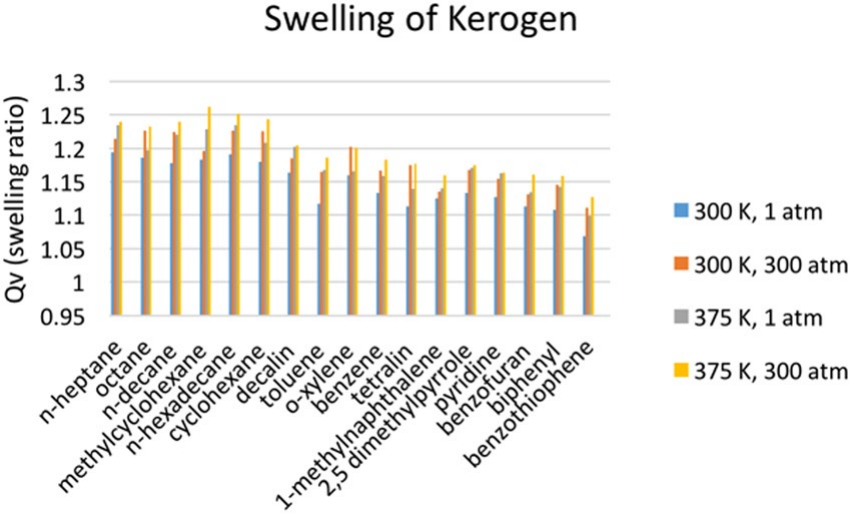
The annealed kerogen matrix swelled while performing NPT ensemble molecular dynamics simulations with a series of fluids as shown here. The swelling ratios of kerogen of Type II (medium to high maturity) in presence of 17 liquids at different temperatures and pressures are shown in the bar graph. The kerogen swells more at higher temperatures and at high pressures.

MD simulations were performed to investigate the swelling of kerogen with various liquids. Each simulation consists of a matrix of kerogen and a liquid, with the weight of liquid equal to the 20% of the weight of the kerogen. It should be noted that all test-tube swelling experiments, on the other hand, are always conducted with excess liquids. In these test-tube experiments, the liquids with higher solubility in kerogen existed in larger amounts in the kerogen and therefore, swelled the kerogen more. The previous test-tube swelling experiments^3,4,7–9,47,48^ conducted with excess liquids show that both types I and II kerogens are characterized by different volumetric swelling ratios for different organic liquids. The MD swelling simulations conducted with the same mass of liquids of different nature (aromatic or aliphatic, heavier or lighter) also show different swelling ratios for type II kerogen (Fig. 11). Thus, the MD simulations performed in this work revealed the differential swelling of type II kerogen by same mass of different hydrocarbon liquids. It was seen in previous test-tube swelling experiments that the heavier polar liquids show more swelling of kerogen since they are the fractions that are preferentially sorbed more than other lighter saturated liquids. On the other hand, such a trend was not observed in MD swelling simulations as the swelling simulations were performed with a fixed mass of each liquid. MD simulations reveal that the same mass of saturates and lighter compounds swell kerogen more than the polar and heavier compounds (Fig. 11). The trend from MD may match experimental results if excess amounts of liquids were used but this remains to be demonstrated. The determination of swelling ratio from MD simulations with excess liquid is not possible as there is no way to know if the swollen volume only consists of sorbed liquid and not free condensed excess liquid volume. In case of 20% liquid saturation by weight, it is safe to assume that all liquid is sorbed into the kerogen matrix as TGA performed on decane-swelled kerogen and pyridine-swelled kerogen reveal that 68% and 84% of the mixture was sorbed decane and sorbed pyridine, respectively. Thus, 20% saturation is well within the threshold beyond which condensed liquid may separate. When the excess amounts of liquids are used, the kerogen absorbs preferred (heavier, polar, aromatic) liquids more and subsequently exhibits greater swelling. This behavior is not replicated in MD simulations that use identical masses of hydrocarbon components. Thus, the same mass of different liquids swell kerogen differently based on MD simulations. The earlier test-tube swelling experiments performed in several previous studies revealed that excess amount of liquids swell kerogen to different extents. It is clear from either or both of MD simulations and test-tube experiments, that the different liquids swell kerogen (of both types) differently (differential swelling of kerogen by different liquids) as also indicated in a previous work^5^.

If kerogen is in contact with oil (a multi-component mixture of hydrocarbons), kerogen would preferentially uptake some components of oil more than others. The overall ability of an oil to swell kerogen is determined by the contributions of the various components that make up the oil. The swelling MD simulations allow the swelling of kerogen to be calculated with different liquids and gases at higher pressures which are difficult to achieve in the laboratory. The swelling of kerogen at high system pressure (300 atmospheres) in MD simulations would resemble the *in-situ* shale reservoir conditions of some of the US shale plays with pore pressure in that range. The MD simulations reveal that the swelling of kerogen increases slightly with increasing temperature and with increasing pressure. The swelling ratios (=volume of swollen kerogen/original volume) increase with pressure because the volume of unswollen kerogen decreases more with higher pressure than the volume of swelled kerogen as liquids fill up the pore spaces in swelled kerogen and do not allow that much decrease in the volume of the kerogen.

## Discussion and Conclusion

The swelling of kerogen has key implications on the expulsion of fluids from kerogen as well as on the original-hydrocarbon-in-place. Both type I and type II kerogen exhibit swelling to different extents with different liquids according to test-tube swelling experiments performed on type I in this work and based on several of the previous studies^3–11^ on swelling of both type I and type II kerogen. Experiments using analytical tools such as DSC and TGA were performed in this work to understand the effect of swelling (differential uptake/preferential sorption) of kerogen and confinement of fluids in organic nano pores, on thermodynamic properties of fluids in shales. The concept of preferential sorption of some components of oil by kerogen has been noted earlier for type I and type II kerogen using Flory Rehner Theory and experiments in previous studies^3,4,7–9,47–49^. The MD simulations performed in this work for type II kerogen show that for the same mass of various liquids, the swelling ratio of kerogen varies from one liquid to other. MD simulations revealed that kerogen swells differently with each liquid. This shows that the different components in a multi-component liquid such as an oil are going to sorb in kerogen to different amounts leading to the overall observed swelling behavior (Qv).

The swelling in type I kerogen is observed with Clay Basin oil. Assuming Clay Basin oil as a multicomponent mixture of hydrocarbons, preferential sorption of heavier, polar, aromatic components of oil by kerogen would change the composition of sorbed phase, thereby partitioning the oil into a sorbed and free oil phase^5,12,43–45^. Such heavier, polar, aromatic rich composition of the sorbed oil will change the bubble point of the sorbed oil when compared to the bubble point of the original oil or free oil. The lighter, saturates rich free oil fraction that was not sorbed into the kerogen matrix would stay in the nano pores (17.7 nm mean radii) and undergo effect of confinement^13–22^ due to dominant pore-wall – fluid interactions^23^. The confinement will again change the bubble point of the free oil fraction. The DSC experiments performed on Clay Basin oil and kerogen swelled by it revealed the combined effect of preferential sorption by kerogen and effect of confinement. A wider peak of the kerogen-oil sample in DSC as opposed to sharper peak for oil also indicates that there are two effects that suppressed the bubble point of oil in Fig. 8. Clay Basin oil sorbed in kerogen has bubble point which is suppressed by around 50 °C from the original bubble point of pure oil. The DSC experiments on decane and decane-swelled kerogen, as well as pyridine and pyridine-swelled kerogen, revealed only the effect of confinement as the sorbed and free fraction partitioned by preferential sorption by kerogen are both decane or pyridine, respectively. Hence, only the effect of confinement caused the suppression of about 7.8 °C in the boiling point temperature of decane when it was with kerogen compared to the boiling point of pure decane. Similarly, the boiling point of sorbed pyridine in pyridine-swelled kerogen was elevated by only 7.5 °C. The TGA data shows that the pyridine was sorbed more in kerogen than decane as it is preferred more in kerogen being a polar, aromatic liquid. However, an important finding is that polar liquid such as pyridine’s boiling point was elevated after being sorbed in kerogen, in contrast to the suppression seen in the boiling point of decane in kerogen. This probably happened because preferred liquid such as pyridine is held stronger in kerogen than saturates like decane. This observation is consistent with the fact that pyridine has larger solubility^5^ (solubility parameter of 21.9 (J/cm^3^)^1/2^) compared to that of decane (15.8 (J/cm^3^)^1/2^) as well as a smaller molar volume^5^ (80.6 cm^3^) compared to that of decane (195.9 cm^3^).

In conclusion, all kinds of swelling kerogens (type I and type II) preferentially uptake lighter, polars and aromatics components of oil more than other and exhibit an overall unique swelling calculated in terms of Q_v_. A Q_v_ of 1.6 was observed in kerogen of type I swelled with Clay Basin oil. Hence, kerogen behaves as a fractionating medium. This fractionation of oil by kerogen splits oil into a sorbed and a free phase. The sorbed phase stays in the kerogen matrix while the free phase may stay in the nano pores of kerogen.

In the current work, it was shown that the effect of preferential sorption by kerogen and confinement in nano pores of kerogen change the boiling point temperatures of hydrocarbon fluids in contact. The effect of kerogen partitioning appears to be more dominant. Before implementing the findings of this work at the field scale, the combined effect of kerogen and confinement should be assessed for the whole shale sample instead of just kerogen as shale consists of pores in the inorganic components (minerals) as well. The change in bubble points has implications on pressure-volume-temperature (PVT) properties of *in-situ* oil (sum of sorbed and free phase oil) in the kerogen-rich shales. The correct bubble points are essential in predicting the true rates and volumes of recoveries from shale plays and must be calculated by accounting for the two effects discussed here. Shifts in the bubble points may delay gas production in liquid rich shale plays and would allow provide improvements in the liquid recovery.

## Methods

### Experimental

Kerogen was isolated from the whole rock shale sample by a series of acid treatments using methods described in previous kerogen isolation experiments^26–30,50^. First, the carbonate minerals are removed by treating 2.5 g of shale rock with 30 ml of 5N HCl for 48 hours. The remaining 1.0 g of the residual rock is treated with 10 ml of 12N HCl and washed with 10 ml of DI water. The residual rock was heated in a vacuum and was ready for the next step of removal of pyrite minerals. To remove the pyrite minerals from the residual rock, it was treated with 20% (by weight) HNO_3_ for 48 hours followed by a wash with DI water and oven dry. The dry residual rock was then treated with 5N HCl followed by 25 ml of 48% (by weight) HF for 48 hours. The residual was washed with DI water followed by drying in an oven. The weight of the rock at each step was documented. In order to calculate the amount of organic and inorganic matter left in the residual rock, thermo gravimetric analysis (TGA) was performed in the residual rock sample. The TGA data revealed that over 75% of the residual sample (powder form) is organic. The experiments with kerogen and kerogen-decane binary mixture were designed using a differential scanning calorimeter (DSC) and thermogravimetric analyzer (TGA). First, 60 mg of kerogen sample was dried at 105–110 °C for 1 hour in an atmospheric oven and cooled overnight in a vacuum oven before performing the swelling experiments. Three samples of 10 mg kerogen each were treated with excess decane in a mini-centrifuge tube while other three were packed in air-tight tubes. The tubes with kerogen-decane were centrifuged at 6000 rpm for three cycles of five-minute duration each. The mixture was kept in a flat bottom test-tube. The initial height of kerogen in the tube was measured by Vernier calipers (with accuracy of ± 0.02 mm) after the third centrifuge (h1). The sample was left for 24 hours and the sample height is measured again (h2). Calculation of swelling from test tube method was calculated as Q_v_ = h2/h1. Similar procedure was also performed for test-tube swelling experiments with Clay Basin oil and pyridine. The three decane-swelled kerogen samples were then used for further experiments. Differential scanning calorimetry and thermogravimetric analysis were performed for the original kerogen and swollen kerogen to measure the change in the boiling point of sorbed decane. Dynamic Light Scattering for particle size analysis was performed on original and swollen kerogen and the results were compared. The DLS experiments were performed with a Wyatt Corporation Dynapro Nanostar^TM^ with a red laser of 658 nm. It can detect sizes in range of 0.2 nm to 2500 nm, and can see particle concentrations as low as 0.1 mg/mL. The original and swollen kerogen particles (swelled for 24 hours with decane) were dispersed in decane immediately before running the samples in DLS. Special care was taken to perform experiments just after preparing the sample particles in order to disperse the particles and avoid any agglomeration that can lead to an error. The test-tube swelling and DLS experiments for all liquids were performed at room temperature and pressure.

For the differential scanning calorimeter, excess liquid layer in tubes that was accumulated over the swollen kerogen bed was removed and the sample was transferred to the DSC pans. Weight of the empty DSC pan was measured. Experiments were performed under atmospheric pressure with an inert gas (nitrogen) flow rate of 40 mL/min. Before each measurement, the DSC cell was purged with the inert gas (nitrogen) for at least 10 min. Hermetically sealed and pierced samples were cooled to −120 °C at a rate of 5°C/min, equilibrated at −120 °C for 10 min, and then heated to 200 °C. At 200 °C, the temperature remained constant for 10 min before stopping the run. This procedure was repeated to run DSC for pure decane (to compare with sorbed decane). Similarly, the kerogen and kerogen-decane samples were analyzed in a thermogravimetric analyzer. The samples were treated at a ramp-rate of 5 °C/min and the weight of the samples was lost as the temperature was increased. A weight loss curve was obtained from the thermogravimetric data. The whole procedure of DSC was repeated again for kerogen and Clay basin oil samples as well as for kerogen and pyridine samples.

### Modeling

**Figure 12:**
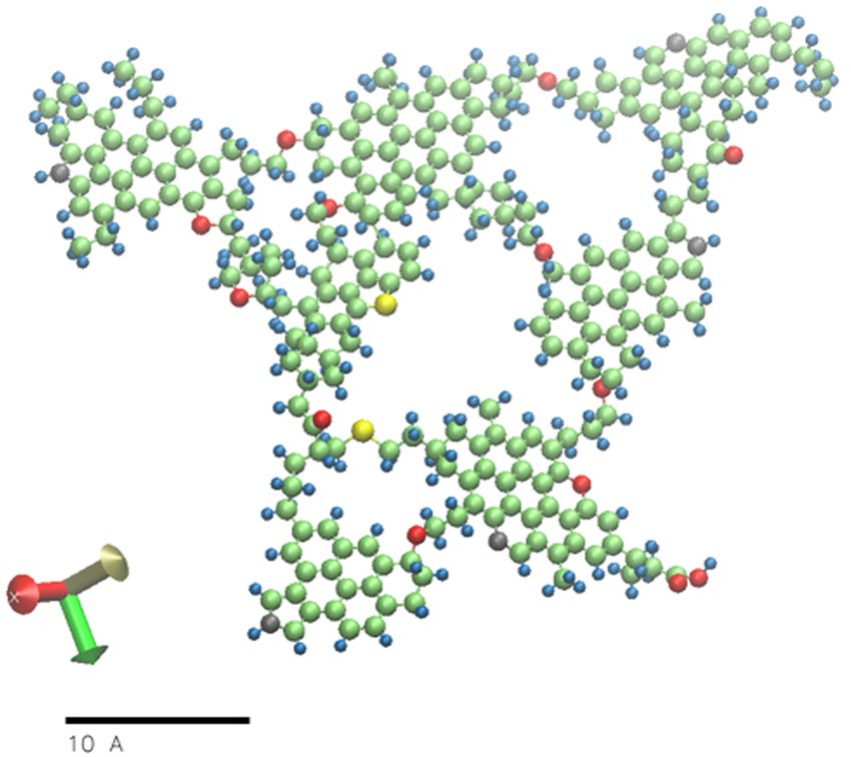
Single molecule of Type II kerogen taken from Ungerer P. *et al*.^46^. The chemical formula of this kerogen model is C_242_H_219_O_13_N_5_S_2_. The color scheme is carbon – green, hydrogen – blue, oxyzen – red, nitrogen – grey and sulphur – yellow.

**Figure 13:**
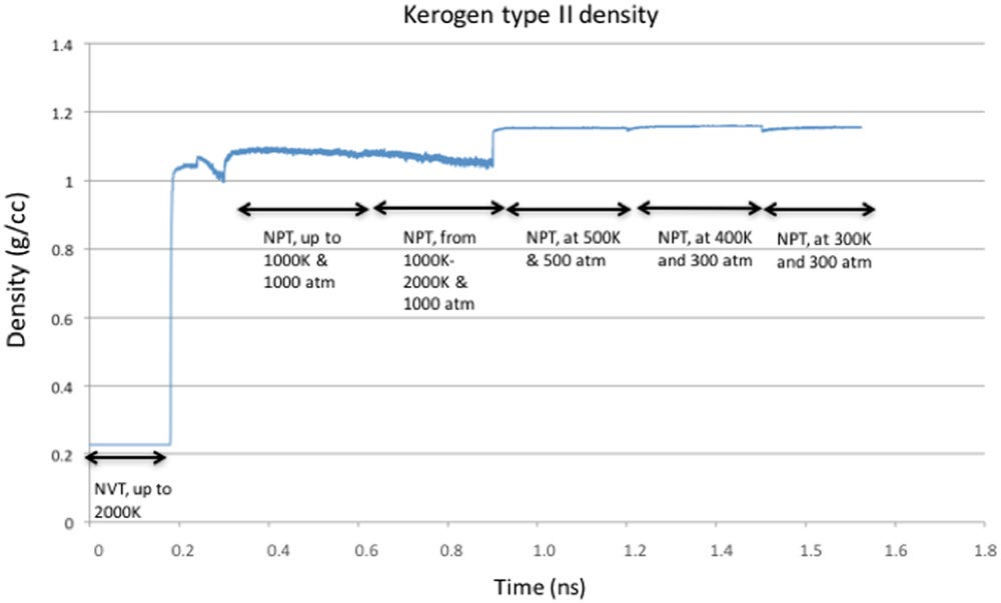
Density of type II kerogen calculated from Molecular Dynamics Simulations. After simulated annealing of 1.6 ns (0.2 ns of NVT ensemble and multiple steps of NPT ensemble of total time 1.4 ns), density of kerogen was around 1.18 g/cc.

MD simulations were for performed to calculate swelling ratios of type II kerogen with a series of hydrocarbons liquids. The details about selection of simulation matrix and MD simulations are discussed in the supplementary information. The current modeling work involves using xyz coordinates of atoms in one of the published kerogen models^46^ (Fig. 12) and remodeling it by assigning parameters from the General Amber Force Field (GAFF)^51^. The bulk kerogen was modeled by relaxing 10 kerogen molecules using systematic ‘Simulated Annealing Procedure’ discussed in the supplementary information. A relaxed bulk kerogen model, in its native state, obtained by simulated annealing procedure was then used to determine the density of bulk kerogen and probability distribution function of the C-C bond lengths. During the course of annealing, the system went through multiple energy minimization steps that consist of 0.2 ns of NVT ensemble and 1.4 ns of multiple steps in NPT ensemble as shown in Fig. 13. The density of the kerogen increases and becomes relatively constant when equilibrium has been achieved after ~1.6 ns.

**Figure 14:**
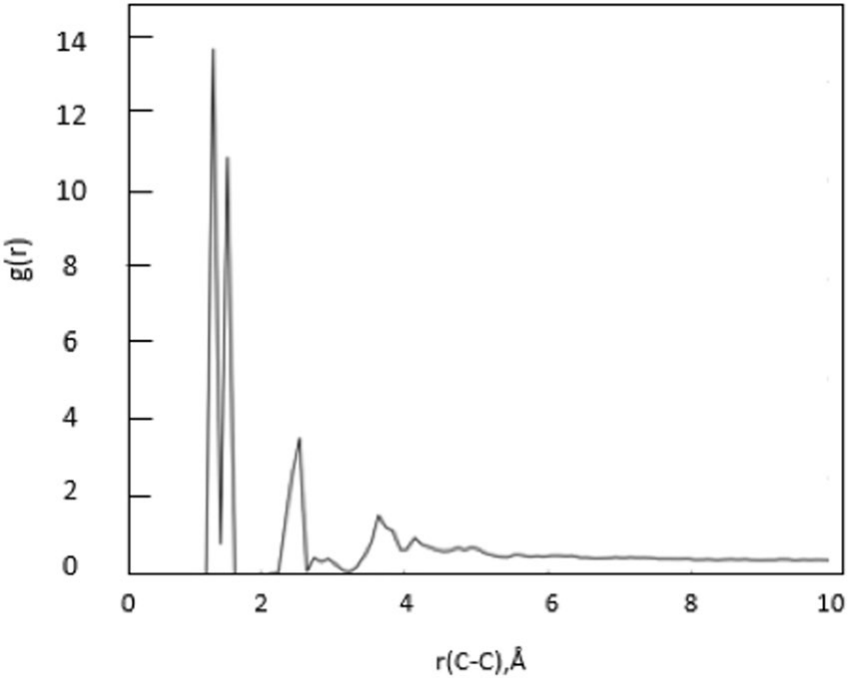
Plot of radial distribution function calculated from type II kerogen molecular model.

Plot of pair-wise radial distribution function (g(r)) and density of the relaxed structure were calculated to validate the kerogen model. A plot of the radial distribution function (Fig. 14) was produced after simulated annealing, during the process of the MD production run at 300 K and 300 atmospheres which matched similar plots for same kerogen model in the previous works^46,53^. The density of annealed structure of modeled kerogen was 1.18 g/cc at 300 K and 300 atmospheres pressure. The density of the type II kerogen after annealing was 1.18 g/cc at 300 K and 300 atmospheres (Fig. 13), which matches well with experimentally measured kerogen densities in range of 1.18-1.23 g/cc reported by Stankiewicz *et al*.^54^ and 1.18-1.25 g/cc by Okiongbo *et al*.^55^.

Once the density and radial distribution function of the kerogen model used in this study are found to be consistent with the results from previous works, kerogen model at its relaxed state was used to measure the extent of swelling of kerogen in presence of different organic liquids. A workflow was developed (discussed in supplementary information) to study the kerogen-hydrocarbons interactions using the GAFF implemented in Large-scale Atomic/Molecular Massively Parallel Simulator (LAMMPS)^52^ tool. The GAFF was originally developed to model organic molecules. Volumetric swelling of kerogen in the presence of organic liquids was studied.

**Figure 15:**
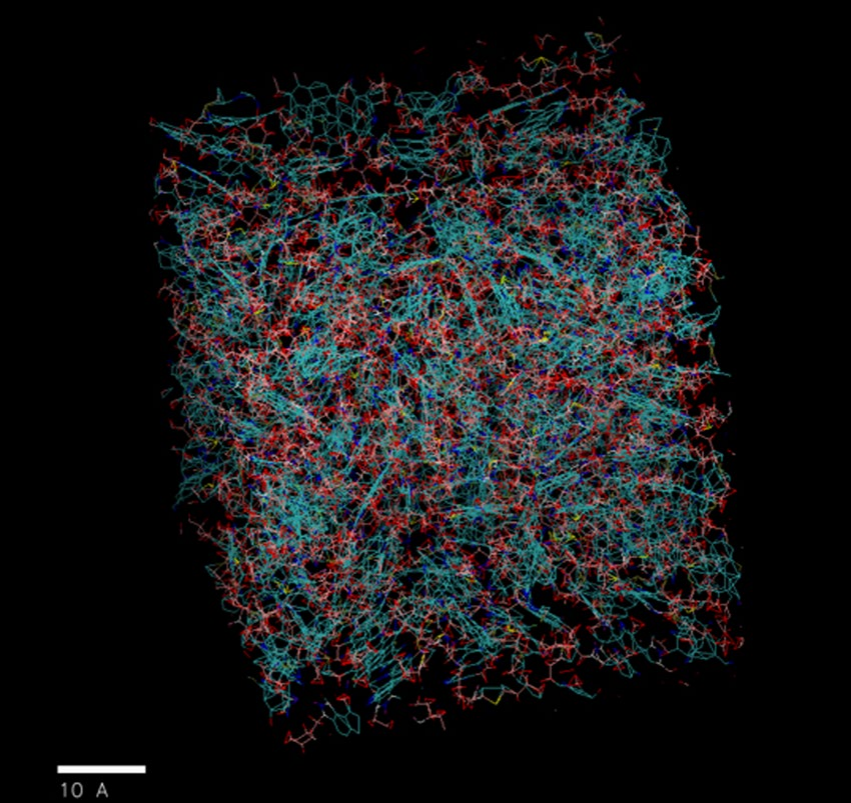
Relaxed structure of kerogen (blue color) and decane (red color) mixture after simulated annealing.

To calculate volumetric swelling (swollen volume/native volume) of kerogen in presence of organic liquids, the kerogen was first annealed together with the liquid molecules till quasi-equilibrium state was achieved. The simulated annealing of kerogen-liquid system was again carried out according to same ‘Simulated Annealing Procedure’ in the supplementary information. After annealing the system to minimize the energy, the MD simulations of kerogen and liquid were performed in isobaric-isothermal (npt) ensemble and periodic boundary conditions. The weight of liquid in the simulation cell (10 nm cube) is equal to 20% of the weight of kerogen (10 molecules of kerogen of molecular mass of 3465 Daltons each). The volume and energy of the system were reduced to the minimum at 300 K and 300 atmospheres. To calculate the volumetric swelling ratios (Qv), the final volume of the relaxed system of kerogen and liquid (Fig. 15) at certain temperature and pressure was compared with that of a relaxed system of pure kerogen.

## Electronic supplementary material

Supplementary Information
