# Supplementary material for: Kerogen Swelling and Confinement: Its implication on Fluid Thermodynamic Properties in Shales
Source: Sci Rep. 2017 Oct 2;7:12530. doi: 10.1038/s41598-017-12982-4 (PMC5624957; doi:10.1038/s41598-017-12982-4)
Supplement: Supplementary file 1 — Supplementary Information [file 41598_2017_12982_MOESM1_ESM.pdf]

Kerogen Swelling and Confinement: Its implication on Fluid Thermodynamic Properties in Shales

Manas Pathak[1] Hyukmin Kweon[1] Milind Deo[*,1] and Hai Huang[2]
[1]University of Utah
[2]Idaho National Laboratory

*Milind.deo@utah.edu

Supplementary Information

Initial Configuration and Matrix Selection in Molecular Dynamics Simulations

The simulation matrix was selected in order to perform simulation of swelling of kerogen with fixed masses of a series of hydrocarbon liquids that ranged from being polar, non-polar, saturates, and aromatic compounds. Thus, a model of type II kerogen was simulated with 17 different liquids in multiple MD simulations. Each simulation consists of a fixed mass of kerogen simulated with a liquid (mass of liquid equals 20% of mass of the kerogen). The kerogen and liquid molecules were randomly inserted in the simulation box and the system was annealed together using the 'Simulated Annealing' procedure discussed later. Initial configuration consists of the liquid molecules placed outside of the kerogen in a simulation box. The liquid molecules diffused into the kerogen and were sorbed by it during the subsequent MD simulations. The properties of the final system at the end of a successful simulation annealing procedure are not a function of the time when the liquid molecules are inserted in the simulation box.

Workflow for Simulated Annealing and Molecular Dynamics Simulations

A method has been developed to construct a molecular structure of bulk kerogen from a single molecule. A simulated annealing procedure was performed on a group of several kerogen molecules using the GAFF force field, which was originally developed for molecular dynamics simulations of organic molecules[1]. In the current work, the application of GAFF was extended from simpler organic molecules to complex organic macromolecules like kerogen. The type II C kerogen model was chosen from Ungerer et al. (2015)[2]. The chemical formula of Model II C is $C_{242}H_{219}O_{13}N_5S_2$ with 481 atoms in one molecule. The H/C and O/C ratios of this model unit are 0.905 and 0.054 respectively, and its location on a Van Krevelen diagram, near the end of the oil window is shown in Figure 1.

The force field parameters for GAFF were chosen from Wang et al, 2004[1] and the 'gaff.lt' file available in the Moletemplate[3] software distribution. The Gasteiger-Marsili[4] charges were assigned to all the atoms using Avogadro molecular modeling tool[5].

In order to perform MD simulations, a workflow was developed comprising of series of steps as shown in figure S1. The steps are discussed below and in figure S1:
1. First, a molecule of Type II C kerogen was converted from (x,y,z) format to protein data bank (PDB) format. A single kerogen model II C is shown in figure 10.
2. The atomtype program of Ambertools[6] was used to convert the file into GAFF friendly PDB format
3. The Packmol (Martinez L, 2009) tool was used to randomly stuff 35 of these kerogen molecule models into a simulation box
4. The Visual Molecular Dynamics, VMD[7] tool was used to produce an input file for LAMMPS[8]
5. The bond, angles and dihedral angle were manually entered for all the atoms of kerogen from gaff.lt
6. An instruction file for LAMMPS, which included the series of simulated annealing steps was written
7. The simulations for simulated annealing, followed by production runs were run in the LAMMPS in parallel with multiple processors of a super computer
8. The density of kerogen and probability distribution function of the C-C bond lengths were calculated at the end of simulated annealing (after relaxation steps).

9. Swelling simulations started with first annealing the bulk kerogen with organic liquid (of weight equal to 20% of that of kerogen) till density becomes constant. VMD was used to visualize the final relaxed structure of pure kerogen and kerogen with liquid (Figure 15) at the end of MD simulation following the annealing steps.

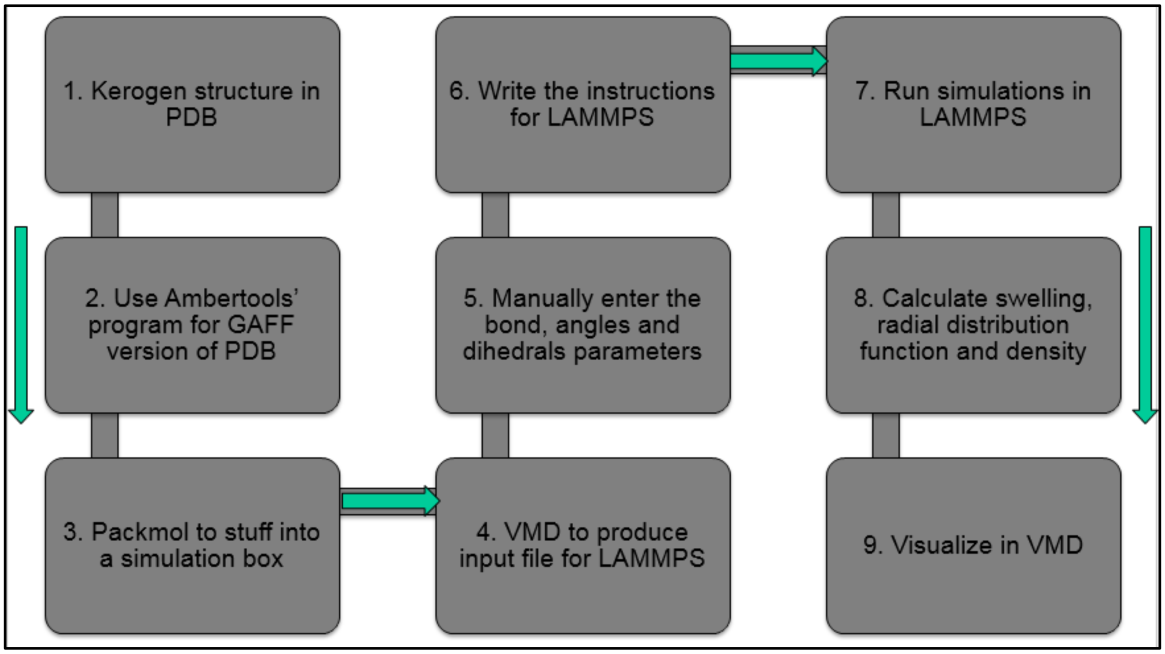

Figure S1: Workflow for modeling kerogen by implementing GAFF force field in LAMMPS

The General Amber Force Field (GAFF) force field was used to calculate the parameters for bonded as well as non-bonded interactions. The 12-6, Lennard-Jones (L-J) potential[9]

$$\emptyset(r)_{L-J} = 4\epsilon_{ij}\left[\left(\frac{\sigma_{ij}}{r_{ij}}\right)^{12} - \left(\frac{\sigma_{ij}}{r_{ij}}\right)^{6}\right] \text{--------------------(1)}$$

was used to calculate the non-bonded molecular forces. Here, ϵ is the depth of the potential well, σ is the finite distance at which the inter-particle potential is zero, r is the distance between the particles. The Lorentz-Berthelot classical mixing rule [10,11] was used to calculate the parameters for unlike atoms in the L-J potential.

$$\sigma_{ij} = \left(\sigma_{ii} + \sigma_{jj}\right)^{2} \text{--------------------(2)}$$

$$\epsilon_{ij} = \sqrt{\epsilon_{ii}\epsilon_{jj}} \text{ --------------------(3)}$$

Gasteiger-Marsili [4] charges were assigned to all the atoms in the kerogen and gas molecules (methane and carbon dioxide) to calculate the coulombic interactions

$$\emptyset(r)_{coulombic} = \frac{C\,q_1 q_2}{\epsilon r} \text{--------------------(4)}$$

Here, q_1 and q_2 are the charges on interacting atoms, ∈ is the dielectric constant of the medium and C is a constant.

Simulated Annealing Procedure

In order to relax the system, the bulk kerogen structure made up of fixed number of kerogen molecules systematically went through series of energy minimization steps. The initial density of kerogen in the 100Å cube was 0.206 g/cc. First, the structure was simulated in a canonical (NVT) ensemble as the temperature was increased to

2000K, during a total time of 0.2 ns (in 2 million time steps) with density of 0.22 g/cc. The system was then simulated in a series of isothermal-isobaric (NPT) ensembles for a total time of 1.4 ns, consisting of 5 smaller steps of 0.28 ns each with step-wise reduction in temperatures and pressures. Each of these steps consist of MD simulations in NPT ensemble with pressure, temperature in each step as (1000K, 1000atm), (1000K-2000K, 1000atm), (500K, 500atm), (400K, 300atm), (300K, 300atm) respectively. These temperatures and pressures during each step is indicated in Figure 13 of the manuscript. The final pressure and temperature was close to reservoir pressure and temperature in order to replicate the realistic in-situ conditions for kerogen. A schematic of all the annealing steps are given in Figure S1. For any of the NPT or NVT cycle, the time steps varied from 0.0001 to 0.01 ns. The density of the final annealed structure of bulk kerogen at minimum energy state was calculated at the end of the simulated annealing procedure.

A Nose-Hoover temperature thermostat and Nose-Hoover pressure barostat were used in LAMMPS molecular simulator to generate positions and velocities sampled from the canonical (NVT) and isothermal-isobaric (NPT) ensembles. Since the pressure includes a kinetic component due to particle velocities, both thermostatting and barostatting in MD simulations require calculation of the temperature. A target temperature (T) and/or pressure (P) mentioned in former paragraph was specified in each run, and the thermostat or barostat attempted to equilibrate the system to the requested T and/or P. The thermostatting and barostatting is achieved by adding some dynamic variables which are coupled to the particle velocities (thermostatting) and simulation domain dimensions (barostatting).

The kerogen-liquid simulations were carried by randomly inserting kerogen and liquid molecules in a 10nm simulation cubic-box. Simulated annealing procedure discussed above was used to first minimize the energy of the system. MD simulations were then performed in NPT ensemble to measure the volume of the system after quasi-equilibrium was reached. The initial configuration of kerogen would have some effect on density of kerogen[12,13] and therefore, on swelling ratios (Qv) calculated by MD simulations. However, this work focused more on the comparison of the Qv for each liquid than the absolute value of Qv for each liquid, i.e. the interest was in obtaining a trend in the swelling ratios of kerogen with different liquids.

References

1. Wang, J., Wolf, R. M., Caldwell, J. W., Kollman, P. A. & Case, D. A. Development and testing of a general amber force field. *J. Comput. Chem.* **25,** 1157–74 (2004).
2. Ungerer, P., Collell, J. & Yiannourakou, M. Molecular Modeling of the Volumetric and Thermodynamic Properties of Kerogen: Influence of Organic Type and Maturity. *Energy & Fuels* **29,** 91–105 (2015).
3. Jewett, A. I., Zhuang, Z. & Shea, J.-E. Moltemplate a Coarse-Grained Model Assembly Tool. *Biophys. J.* **104,** 169a (2013).
4. Gasteiger, J. & Marsili, M. Iterative partial equalization of orbital electronegativity—a rapid access to atomic charges. *Tetrahedron* **36,** 3219–3228 (1980).
5. Hanwell, M. D. *et al.* Avogadro: an advanced semantic chemical editor, visualization, and analysis platform. *J. Cheminform.* **4,** 17 (2012).
6. Case, D. A. *et al.* The Amber biomolecular simulation programs. *J. Comput. Chem.* **26,** 1668–1688 (2005).
7. Humphrey, W., Dalke, A. & Schulten, K. VMD: visual molecular dynamics. *J. Mol. Graph.* **14,** 33–8, 27–8 (1996).
8. Plimpton, S. Fast Parallel Algorithms for Short-Range Molecular Dynamics. *J. Comput. Phys.* **117,** 1–19 (1995).
9. Jones, J. E. On the Determination of Molecular Fields. II. From the Equation of State of a Gas. *Proc. R. Soc. A Math. Phys. Eng. Sci.* **106,** 463–477 (1924).
10. Berthelot D. C. R. No Title. *Hebd. Seanc. Acad. Sci., Paris* **126,** 1703. (1898).
11. Lorentz, H. A. Ueber die Anwendung des Satzes vom Virial in der kinetischen Theorie der Gase. *Ann. Phys.* **248,** 127–136 (1881).
12. Zhao, T., Li, X., Zhao, H. & Li, M. Molecular simulation of adsorption and thermodynamic properties on type II kerogen: Influence of maturity and moisture content. *Fuel* **190,** 198–207 (2017).

13. Ho, T. A., Criscenti, L. J., Wang, Y., Grest, G. S. & Yang, R. S. Nanostructural control of methane release in kerogen and its implications to wellbore production decline. *Sci. Rep.* **6,** 28053 (2016).
